# Advanced Top-Down Fabrication for a Fused Silica Nanofluidic Device

**DOI:** 10.3390/mi11110995

**Published:** 2020-11-09

**Authors:** Kyojiro Morikawa, Yutaka Kazoe, Yuto Takagi, Yoshiyuki Tsuyama, Yuriy Pihosh, Takehiko Tsukahara, Takehiko Kitamori

**Affiliations:** 1 Department of Applied Chemistry, School of Engineering, The University of Tokyo, 7-3-1 Hongo, Bunkyo-ku, Tokyo 113-8656, Japan; kazoe@sd.keio.ac.jp (Y.K.); takagi@icl.t.u-tokyo.ac.jp (Y.T.); pihosh_y@enesys.rcast.u-tokyo.ac.jp (Y.P.); 2 Department of Bioengineering, School of Engineering, The University of Tokyo, 7-3-1 Hongo, Bunkyo-ku, Tokyo 113-8656, Japan; tsuyama@icl.t.u-tokyo.ac.jp; 3 Laboratory for Advanced Nuclear Energy, Institute of Innovative Research, Tokyo Institute of Technology, 2-12-1-N1-6, Ookayama, Meguro-ku, Tokyo 152-8550, Japan; ptsuka@lane.iir.titech.ac.jp

**Keywords:** nanofluidics, nanochannel, micro-nano interface, nanofabrication, top-down fabrication, lab-on-a-chip, streaming current

## Abstract

Nanofluidics have recently attracted significant attention with regard to the development of new functionalities and applications, and producing new functional devices utilizing nanofluidics will require the fabrication of nanochannels. Fused silica nanofluidic devices fabricated by top-down methods are a promising approach to realizing this goal. Our group previously demonstrated the analysis of a living single cell using such a device, incorporating nanochannels having different sizes (10^2^–10^3^ nm) and with branched and confluent structures and surface patterning. However, fabrication of geometrically-controlled nanochannels on the 10^1^ nm size scale by top-down methods on a fused silica substrate, and the fabrication of micro-nano interfaces on a single substrate, remain challenging. In the present study, the smallest-ever square nanochannels (with a size of 50 nm) were fabricated on fused silica substrates by optimizing the electron beam exposure time, and the absence of channel breaks was confirmed by streaming current measurements. In addition, micro-nano interfaces between 10^3^ nm nanochannels and 10^1^ μm microchannels were fabricated on a single substrate by controlling the hydrophobicity of the nanochannel surfaces. A micro-nano interface for a single cell analysis device, in which a nanochannel was connected to a 10^1^ μm single cell chamber, was also fabricated. These new fabrication procedures are expected to advance the basic technologies employed in the field of nanofluidics.

## 1. Introduction

Nanofluidics is the study of fluids confined in nanochannels, and has recently attracted much attention with regard to the development of new functionalities and applications. The primary feature of the nanospaces involved in this field is their small size, since spaces on the nanometer scale are similar to the size of macromolecules. The volumes of these nanospaces are on the attoliter (10^−18^ L) to femtoliter (10^−15^ L) scale, which is many times smaller than the volumes of liquids handled in conventional bulk techniques. In addition, since the surface-to-volume ratio of a nanospace is extremely high, the surface is its dominant feature. The surface charges on a nanochannel can form an electric double layer in the liquid phase with dimensions on the order of 10^0^–10^2^ nm, which is close to the size of the channel itself. In such nanospaces, chemical properties such as ion concentrations can become heterogeneous even when uniform in the bulk liquid. Our group previously reported that the properties of the liquid are also different in nanospaces compared with those in the bulk liquid [1,2]. Therefore, the unique aspects of nanospaces (small dimensions, extremely low volumes, very high surface-to-volume ratios and unique liquid properties) can be used to realize novel functional devices that are difficult to obtain using conventional bulk spaces.

A key issue related to the fabrication of nanofluidic devices is the construction of nanochannels. Many nanofluidic devices are based on nanopores [3,4,5] or nanotubes [6,7,8,9] with dimensions on the 10^0^–10^1^ nm size scale that are fabricated primarily by bottom-up methods, although nanopores on the 10^1^–10^2^ nm scale have also been fabricated by track-etch techniques [10,11,12]. However, there are certain limitations concerning the application of these nanospaces to functional devices. The integration of nanopores/nanotubes with different sizes into one device, the complicated designs that involve branches and confluences, and the patterning of surfaces are all still difficult.

Since the year 2000, the field of nanofluidics has advanced along with the miniaturization of microfluidics [13,14,15,16]. In this approach, nanochannels are fabricated mainly using top-down methods. There are a wide variety of substrate materials such as silicon, fused silica, polymer and so on. Fabrication methods are also various such as lithography, etching, milling, deposition and molding. Using such fabricated nanochannels, many kinds of functional devices were developed. Briefly, DNA manipulation [17,18,19], ion concentration [20,21,22,23], ionic diode [24,25,26], protein detection [27,28,29] were reported. Especially in our group, fundamental technologies for nanofluidics, including nanofabrication processes as well as nanofluidic control and detection techniques have been developed using fused silica nanofluidic devices fabricated by lithography and etching processes [1]. The fused silica nanofluidic device is highly beneficial because fused silica has several advantages, including transparency, mechanical and chemical stability, UV transmittance and facile surface modification. In addition, we have devised processes for the integration of these units that we term micro unit operations (MUOs) and nano unit operations (NUOs). Various MUOs and NUOs, such as mixing, extraction and phase separation, are combined in parallel and in series (similar to an electric circuit) to obtain continuous flow chemical processing (CFCP) [30]. These technologies and methodologies have been employed to fabricate many different functional devices, such as single-molecule ELISA (enzyme-linked immunosorbent assay) devices [31], femtoliter chromatography [32] and picoliter enzyme reactors [33]. In addition, the analysis of a living single cell in a nanofluidic device has been demonstrated [34]. In some of these devices, nanochannels with different sizes (10^2^–10^3^ nm) having branched and confluent structures and with surface patterning have been integrated. Micro-nano interface fabrication is also an important aspect of creating nanofluidic devices. Due to the large size scale differences between nanospaces and bulk, microspaces are required as size interfaces. Microchannels can also serve as single cell chambers in micro/nanofluidic devices for single cell analysis [34,35].

The fabrication of nanochannels on the 10^1^ nm size scale by top-down methods remains challenging. The fabrication of 10^1^ nm channels on fused silica substrates using simple lithography and etching processes has not yet been reported, because there is no information about experimental condition for lithography and etching to fabricate such small nanochannels. Control of nanochannel geometry is also important because the properties of liquids confined in nanochannels will vary based on the geometry (such as square or rectangular shapes) [36]. In addition to nanochannel fabrication, the formation of micro-nano interfaces is challenging. Due to the size scale difference between microchannels and nanochannels, usually microchannels and nanochannels are fabricated by different fabrication methods. For that reason, our group previously fabricated microchannels and nanochannels on two different substrates, and then bonded the substrates such that the nanochannels were connected to the microchannels. To fabricate micro-nano interfaces on a single substrate, some fabrication procedure was considered as follows: initially forming a nanochannel on a substrate, followed by microchannel fabrication using a conventional wet etching method involving hydrofluoric acid. However, this technique would be difficult because the nanochannels could be damaged by the hydrofluoric acid. Limitations such as these therefore hinder the realization of future functional nanofluidic devices. To develop it, combination of microfabrication and nanofabrication is required.

In the present study, our goal was to address the issues discussed above by fabricating geometrically-controlled nanochannels on the 10^1^ nm size scale and micro-nano interfaces on a single fused silica substrate. In this work, the nanochannels were fabricated on a fused silica substrate using electron beam lithography and dry etching while optimizing the electron beam exposure time. Following this, streaming current measurements [37] were performed to confirm successful fabrication of the 10^1^ nm nanochannels. To fabricate micro-nano interfaces on a single substrate, microchannels were subsequently fabricated by dry etching rather than wet etching, so as to prevent damage to the nanochannels. This was accomplished by carefully selecting the fabrication process sequence and optimizing the resist parameters to protect the nanochannels.

## 2. Materials and Methods 

### 2.1. Nanochannel Fabrication

**Figure 1:**
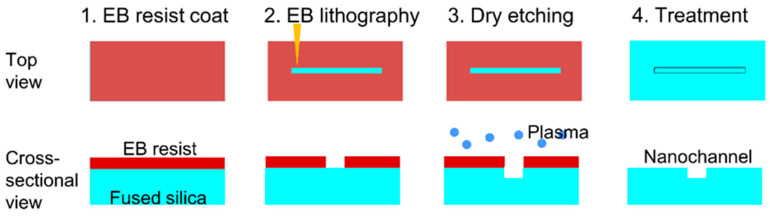
Schematic illustration of the nanochannel fabrication process.

Figure 1 presents a diagram of the procedure used to fabricate nanochannels in this work. In this process, the electron beam resist material ZEP-520A (Zeon Corp., Tokyo, Japan) was diluted threefold with anisole (Zeon Corp., Tokyo, Japan) and spin-coated onto a fused silica substrate (70 mm × 30 mm × 0.7 mm thickness, VIO-SILSX, Shin-Etsu Quartz Co., Ltd., Tokyo, Japan) at 5000 rpm. Electron beam lithography with an ELS-7800 instrument (Elionix, Tokyo, Japan) was subsequently performed while varying the exposure time (0.4, 0.5, 0.6, 0.7 or 0.8 μs/dot; 1 dot = 5 nm × 5 nm area). After electron beam exposure, the resist was developed with *o*-xylene for a time span of 1.5 min, following which the nanochannels were dry etched using an NLD-570 system (ULVAC Co., Ltd., Kanagawa, Japan) with gaseous SF_6_ and CHF_3_. The resist was subsequently removed by washing with a mixture of *o*-xylene and dimethyl sulfoxide, after which the nanochannels were observed by SEM (scanning electron microscopy).

### 2.2. Streaming Current Measurements

In addition to the SEM observations, streaming current measurements were performed to confirm that the nanochannels had no breaks. The details of the experimental setup for these measurements have been provided in previous papers [37]. Briefly, the silanol groups on a fused silica surface will dissociate in an aqueous medium, after which the surface charge and accumulated counter ions in the proximity of the surface will form an EDL (electric double layer). The application of external pressure will generate an ion flow in the EDL and this flow in turn produces a streaming current. If there are breaks in the nanochannels, liquid will not be able to flow through these channels and no streaming current signal will be detected. Using a pressure controller and Ag-AgCl electrodes, streaming currents were measured while varying the applied pressure (100, 200, 300, 350 or 400 kPa).

### 2.3. Micro-Nano Interface Fabrication

**Figure 2:**
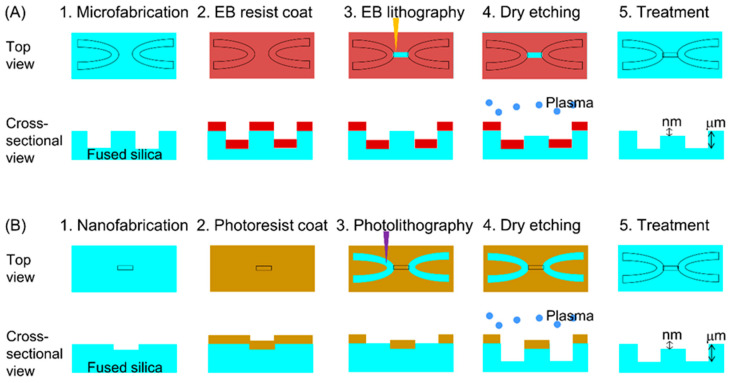
Schematic illustrations of the two micro-nano interface fabrication procedures. (**A**) Microchannels were firstly fabricated, and after that nanochannels were fabricated on the substrate. (**B**) Nanochannels were firstly fabricated, and after that microchannels were fabricated on the substrate.

Two microchannel fabrication procedures were investigated in the present study, as shown in Figure 2. The first procedure is shown in Figure 2A. In this process, microchannels were initially fabricated on the substrate, after which spin-coating of the electron beam resist, electron beam lithography, development of the electron beam resist and dry etching of the nanochannels using SF_6_ and CHF_3_ were performed. The second procedure is presented in Figure 2B. In this procedure, nanochannels were first fabricated on the substrate, following which spin-coating of the photoresist (KMPR^®^ 1035; Microchem Corp., MA, USA) was performed at 3000 rpm, with subsequent photolithography, development of the photoresist and dry etching of the microchannels using gaseous Ar, C_3_F_8_ and CHF_3_ [38]. Before spin-coating, contact angle measurements of the KMPR^®^ photoresist on a substrate were performed to confirm adhesion of this material to the substrate surface. Four types of substrates were prepared for these trials: fused silica, 1,1,1,3,3,3-hexamethyldisilazane (HMDS; Wako Pure Chemical Industries, Ltd., Osaka, Japan) modified fused silica, fused silica with nanochannels (width: 5200 nm and depth: 2100 nm) and HMDS-modified fused silica with nanochannels (width: 5200 nm and depth: 2100 nm). The microchannels and nanochannels fabricated in each procedure were observed by SEM.

## 3. Results and Discussion

### 3.1. Nanochannel Fabrication

**Figure 3:**
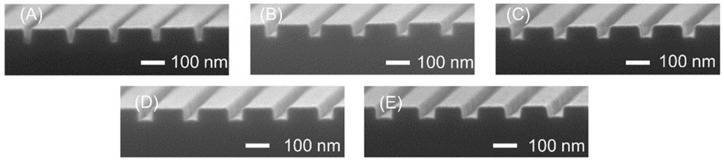
SEM images of nanochannels fabricated using exposure times of (**A**) 0.4, (**B**) 0.5, (**C**) 0.6, (**D**) 0.7 and (**E**) 0.8 μs/dot.

Figure 3 shows SEM images of the fabricated nanochannels. The nanochannels in Figure 3A, fabricated using an exposure time of 0.4 μs/dot, had a triangular shape because the exposure time was not optimized, and some resist was retained in the nanochannel region after development. In contrast, the nanochannels in Figure 3B–E exhibit approximately rectangular shapes. With increases in the exposure time, the nanochannels also became wider, and the optimal exposure time was determined to be 0.5 μs/dot. Using this exposure, nanochannels with dimensions of 48 ± 3 nm (width) and 49 ± 1 nm (depth) with approximately square shapes were obtained. Several groups have demonstrated the formation of 10^1^ nm nanochannels based on lithography and the etching of silicon substrates [39], lithography and molding of PDMS (polydimethylsiloxane) [40,41,42], nanoimprint lithography on polymer substrates [43,44], combinations of lithography, etching and deposition techniques [45,46,47,48], FIB (focused ion beam) milling on fused silica substrates [49,50] and wet etching of fused silica capillary [51]. However, the fabrication of 10^1^ nm channels on fused silica substrates using simple lithography and etching processes has not yet been reported. This is the world smallest geometrically-controlled square nanochannel within a fused silica nanofluidic device using lithography and etching processes.

### 3.2. Streaming Current Measurements

**Figure 4:**
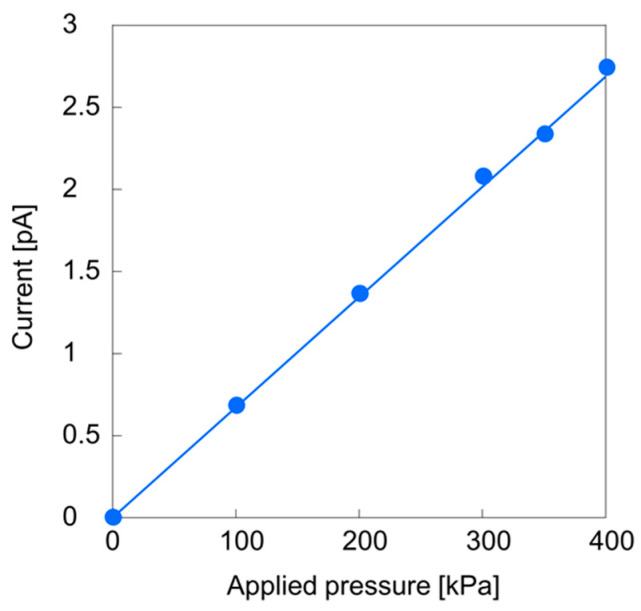
Results of streaming current measurements based on the analysis of 100 nanochannels with dimensions of 48 nm (width), 49 nm (depth) and 200 mm (length).

The streaming currents generated in the 50 nm square nanochannels were examined, with the results shown in Figure 4. These data show a linear increase in the streaming current with increasing applied pressure. This linearity agrees with theoretical predictions and indicates that the nanochannels functioned as fluid channels and did not contain breaks. These specimens represent the smallest-ever square nanochannels formed on a fused silica substrate. Various previously proposed liquid models [1,2] suggest that 50 nm spaces will have a so-called proton transfer phase that exhibits different properties from the bulk liquid. In previous reports for liquids in 10^1^ nm-sized channels using top-down fabricated nanofluidic devices, an electro-osmotic flow [40], water filling motions [42], introduction of fluorescent molecules [43,47,49] and electrical conductance [46,47,48] were observed. The results of the present study are the first demonstration of direct observation of a pressure-driven flow in 50 nm-sized nanochannels within a top-down fabricated nanofluidic device.

### 3.3. Micro-Nano Interface Fabrication

**Figure 5:**
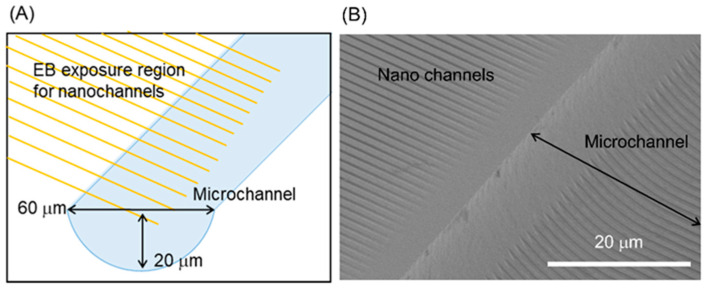
(**A**) Schematic illustration of the area over which nanochannels were fabricated on a substrate incorporating a microchannel. (**B**) SEM image of the resulting nanochannels.

Figure 5 shows the morphology obtained from the procedure in Figure 2A. In this process, a microchannel (width: 60 μm and depth: 20 μm) was first fabricated by wet etching, after which nanochannels (width: 500 nm and depth 200 nm) were formed. The schematic in Figure 5A demonstrates that the electron beam exposure was designed so that the nanochannels would connect with the microchannel. However, as shown in Figure 5B, the nanochannels exhibited gaps near the edge of the microchannel and so were not connected to the microchannel. This effect is attributed to the difficulty in obtaining uniform spin-coating on the substrate when forming the microchannel. Specifically, the thickness of the electron beam resist along the edge of the microchannel was greater than that in other areas, so that the nanochannels did not form properly. 

To address the issues evident in Figure 2A, the procedure in Figure 2B was performed. Before starting the process, the contact angles of the KMPR^®^ photoresist on various substrates were measured to confirm the adhesion of the photoresist to the surface, with the results shown in Table 1. The contact angle on the unmodified substrate was greater than 90°, indicating that the hydrophilic fused silica surface repelled the material. In addition, the contact angle on the nanochannel region of the unmodified substrate was much larger than that on the flat surface. This difference is ascribed to the so-called pinning effect [52,53,54,55] in which a nanostructure enhances the repelling effect. Therefore, the adhesion of the KMPR^®^ photoresist to fused silica surfaces was insufficient, such that the direct coating of the photoresist on the substrates was difficult. The contact angle on HDMS-modified surfaces was found to be lower. In particular, the angle on the nanochannel region of the HDMS-modified substrate was much smaller than that on the nanochannel region without modification. Therefore, to ensure sufficient adhesion of the photoresist, HDMS modification was added to the procedure in Figure 2B.

**Figure 6:**
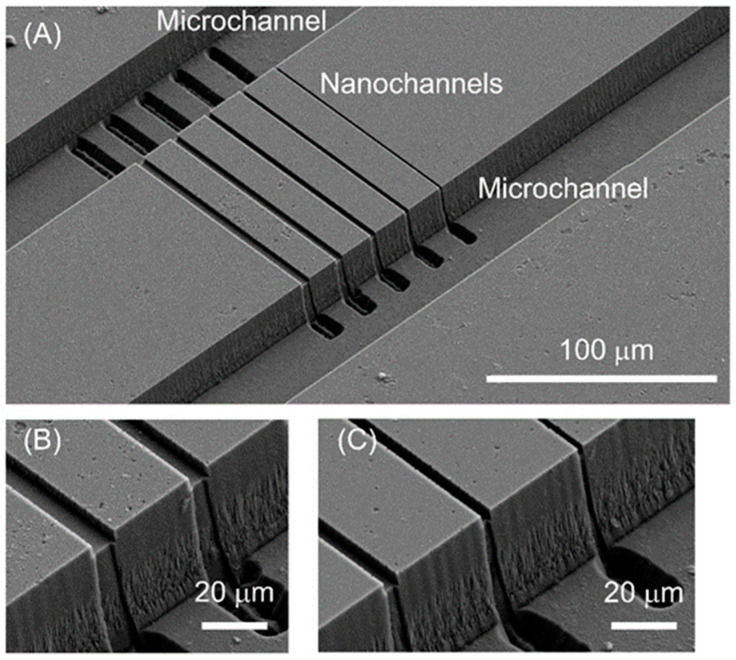
SEM images of microchannels formed on a substrate already having nanochannels. (**A**) Microchannels (width: 51 μm and depth: 23 μm) connected to nanochannels (widths: 1100, 2100, 3100, 4100 and 5100 nm, depth: 2200 nm). (**B**) Enlarged image showing the connections of the 4100 and 5100 nm channels. (**C**) Enlarged image showing the connections of the 1100, 2100 and 3100 nm channels.

**Figure 7:**
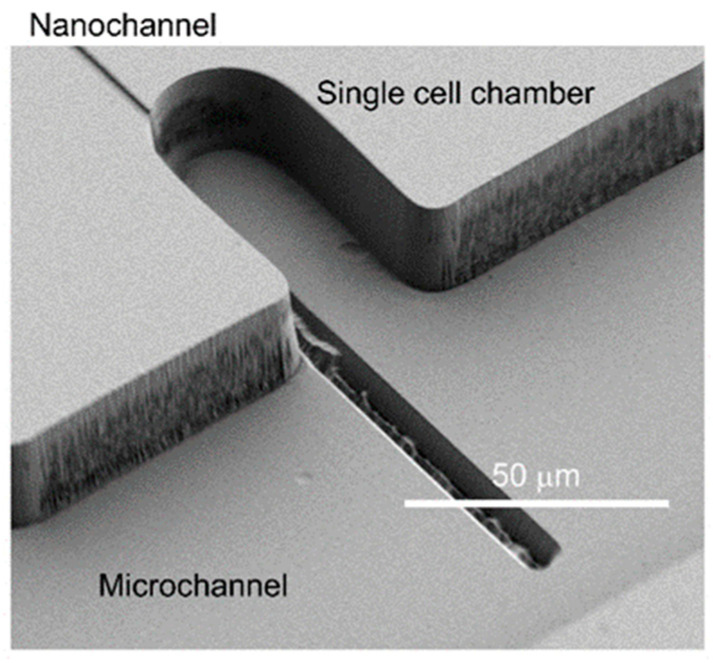
SEM image of the fabricated nanochannel, single cell chamber and microchannel.

The specimens resulting from this modified process are presented in Figure 6. In this procedure, nanochannels were first fabricated, followed by HDMS modification, KMPR^®^ photoresist spin-coating, photolithography, development, and dry etching. As shown in Figure 6A, microchannels (width: 51 μm and depth: 23 μm) connected to individual nanochannels (widths: 1100, 2100, 3100, 4100 or 5100 nm, depth: 2200 nm) were successfully fabricated without damage to the nanochannels. Figure 6B,C provide enlarged images of the connections between the microchannels and nanochannels. These images confirm that the rectangular shapes of the nanochannels were maintained after microchannel etching. Generally, deeper nanochannels are more difficult to uniformly coat with a photoresist and to protect from damage during the microchannel fabrication process. The present results indicate that micro-nano interfaces based on 10^3^ nm nanochannels were successfully formed, suggesting that micro-nano interfaces using nanochannels on the 10^1^–10^2^ nm size scale could be fabricated using the present technique. The micro-nano interface fabrication is principally possible by other fabrication methods such as molding [40], nanoimprint [43] and deposition [47] with different materials than fused silica. In the present study, by using lithography and etching methods which are easy to control channel size and geometry, micro-nano interfaces are fabricated on a single fused silica substrate for the first time. Finally, this fabrication procedure was applied to the fabrication of a living single cell analysis device. As shown in Figure 7, a single cell chamber (width at the entrance: 32 μm, diameter at the curved region: 32 μm and depth: 22 μm) was successfully made and was connected to a nanochannel (width: 2000 nm and depth: 930 nm). These results confirm that the present fabrication procedure can be employed to obtain devices with more complicated structures. In this device, a single cell introduced into the microchannel can be trapped to the single cell chamber by manipulation with an optical tweezer. In the living single cell sampling device [35], the single cell can be connected to the nanochannel and the cytoplasm of the single cell can be introduced into the nanochannel with keeping the single cell alive. In the living single cell device for cytokine analysis [34], the cytokines released in the single cell chamber can be introduced into the nanochannel and can be analyzed in the nanochannel. In our previous reports [34,35], the nanochannel and the single cell chamber were fabricated on two different substrates. In the present study, the nanochannel and the single cell chamber was successfully fabricated on a single substrate, therefore, more functions can be added by combination of fabrications to the other substrate such as other nanochannels fabrication, surface modification with different chemicals, nano-valve [56] structure integration and so on.

## 4. Conclusions

Geometrically-controlled 10^1^ nm nanochannels and micro-nano interfaces were fabricated on fused silica substrates using lithography and etching processes. Although fabrication of them using other fabrication method such as deposition, molding and milling on other substrate materials than fused silica was reported, it was difficult on fused silica substrate by lithography and etching processes. In the present study, precise 50 nm square nanochannels were first fabricated on the fused silica substrate by electron beam lithography and dry etching, employing an electron beam exposure time of 0.5 μs/dot after applying an electron beam resist material that had been diluted threefold. Streaming current measurements confirmed the successful fabrication of the nanochannels without channel breaks, indicating that the smallest-ever square nanochannels on a fused silica substrate had been obtained. The first confirmation a pressure-driven flow of liquids in 50 nm channels within a fused silica nanofluidic device was also directly confirmed. In addition, micro-nano interfaces were fabricated at which 10^3^ nm nanochannels were successfully connected to 10^1^ μm microchannels. In this process, a photoresist was uniformly applied to an HDMS-modified substrate incorporating nanochannels by controlling the hydrophobicity of the nanochannel surfaces. This represents a promising approach to generating micro-nano interfaces using 10^1^–10^2^ nm nanochannels. Finally, a micro-nano interface intended for single cell analysis was fabricated by connecting a nanochannel to a 10^1^ mm single cell chamber. These results are first fabrication of micro-nano interfaces on a single fused silica substrate fabricated by lithography and etching methods. The procedures reported herein are expected to allow the design of new functional devices and to provide new synthetic techniques for nanofluidics.

## Figures and Tables

**Table 1:** Results of contact angle measurements of KMPR photoresists on various surfaces.

KMPR Flat	KMPR Channel	KMPR HDMS Flat	KMPR HDMS Channel
105° ± 12°	134° ± 19°	91° ± 5°	85° ± 5°
